# *trans*-Di­aqua­bis­(pyridazine-3-carboxyl­ato-κ^2^ *N* ^2^,*O*)cobalt(II) dihydrate

**DOI:** 10.1107/S1600536813017340

**Published:** 2013-06-29

**Authors:** Beñat Artetxe, Santiago Reinoso, Leire San Felices, Jagoba Martín-Caballero, Juan M. Gutiérrez-Zorrilla

**Affiliations:** a Departamento de Química Inorgánica, Facultad de Ciencia y Tecnología, Universidad de País Vasco UPV/EHU, PO Box 644, E-48080 Bilbao, Spain

## Abstract

The title compound, [Co(C_5_H_3_N_2_O_2_)_2_(H_2_O)_2_]·2H_2_O, contains a Co^II^ ion on an inversion center, exhibiting an octa­hedral coordination geometry. The equatorial plane is formed by two *trans*-related *N*,*O*-bidentate pyridazine-3-carboxyl­ate ligands and the axial positions are occupied by two water mol­ecules. The Co^II^ complex mol­ecules are stacked in a column along the *a-*axis direction by an O—H⋯N hydrogen bond between the non-coordinating pyridazine N atom and the coordinating water mol­ecule. These columns are further connected into a layer parallel to the *ac* plane by additional hydrogen bonds involving the coordinating and non-coordinating water mol­ecules, and the non-coordinating carboxyl­ate O atom. The crystal packing is completed by inter­layer weak C—H⋯O inter­actions.

## Related literature

For the isotypic zinc(II) and manganese(II) complexes, see: Gryz *et al.* (2003 ▶); Ardiwlnata *et al.* (1989 ▶). For a related zinc(II) complex which does not contain non-coordinating water mol­ecules, see: Gryz *et al.* (2004 ▶).
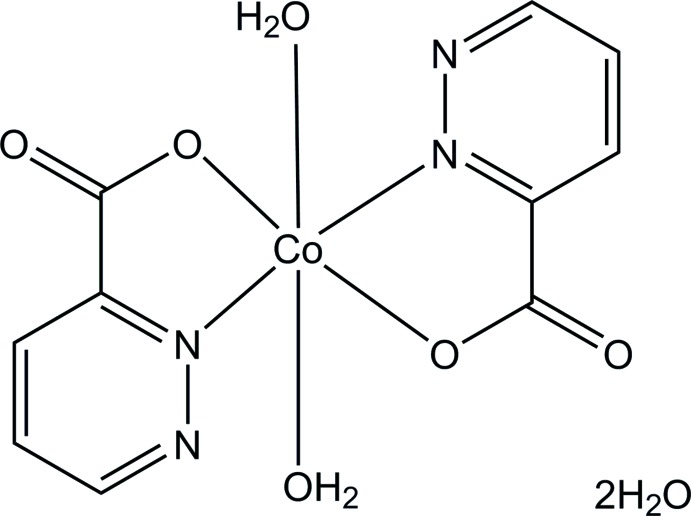


## Experimental

### 

#### Crystal data

[Co(C_5_H_3_N_2_O_2_)_2_(H_2_O)_2_]·2H_2_O
*M* *_r_* = 377.18
Triclinic, 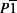

*a* = 5.2934 (4) Å
*b* = 7.2817 (8) Å
*c* = 9.6196 (9) Å
α = 79.673 (8)°
β = 89.875 (7)°
γ = 72.321 (8)°
*V* = 347.01 (6) Å^3^
*Z* = 1
Mo *K*α radiation
μ = 1.29 mm^−1^
*T* = 100 K
0.09 × 0.07 × 0.05 mm

#### Data collection

Agilent SuperNova diffractometer
Absorption correction: multi-scan (*CrysAlis PRO*; Agilent, 2011 ▶) *T*_min_ = 0.907, *T*_max_ = 0.967
2202 measured reflections
1369 independent reflections
1309 reflections with *I* > 2σ(*I*)
*R*_int_ = 0.018

#### Refinement

*R*[*F* ^2^ > 2σ(*F* ^2^)] = 0.026
*wR*(*F* ^2^) = 0.058
*S* = 1.08
1369 reflections
122 parameters
4 restraints
H atoms treated by a mixture of independent and constrained refinement
Δρ_max_ = 0.28 e Å^−3^
Δρ_min_ = −0.31 e Å^−3^

### 

Data collection: *CrysAlis PRO* (Agilent, 2011 ▶); cell refinement: *CrysAlis PRO*; data reduction: *CrysAlis PRO*; program(s) used to solve structure: *OLEX2* (Dolomanov *et al.*, 2009 ▶); program(s) used to refine structure: *SHELXL97* (Sheldrick, 2008 ▶); molecular graphics: *ORTEP-3 for Windows* (Farrugia, 2012 ▶); software used to prepare material for publication: *WinGX* (Farrugia, 2012 ▶) and *PLATON* (Spek, 2009 ▶).

## Supplementary Material

Crystal structure: contains datablock(s) I, global. DOI: 10.1107/S1600536813017340/is5284sup1.cif

Structure factors: contains datablock(s) I. DOI: 10.1107/S1600536813017340/is5284Isup2.hkl

Additional supplementary materials:  crystallographic information; 3D view; checkCIF report

## Figures and Tables

**Table 1:** Selected bond lengths (Å)

Co1—O1	2.0689 (13)
Co1—N2	2.1023 (16)
Co1—O1*W*	2.1199 (14)

**Table 2:** Hydrogen-bond geometry (Å, °)

*D*—H⋯*A*	*D*—H	H⋯*A*	*D*⋯*A*	*D*—H⋯*A*
O2*W*—H2*WA*⋯O2^i^	0.83 (2)	1.96 (2)	2.787 (2)	175 (2)
O1*W*—H1*WA*⋯O2*W*	0.82 (2)	1.92 (2)	2.732 (2)	171 (3)
O2*W*—H2*WB*⋯O2^ii^	0.83 (2)	2.05 (2)	2.865 (2)	168 (3)
O1*W*—H1*WB*⋯N1^iii^	0.82 (2)	2.07 (3)	2.862 (2)	164 (3)
C4—H4⋯O2^iv^	0.95	2.37	3.188 (2)	145
C6—H6⋯O1*W* ^v^	0.95	2.33	3.264 (3)	166

Symmetry codes: (i) 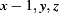; (ii) 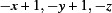; (iii) 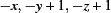; (iv) 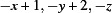; (v) 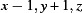.

**Fig. 1.**
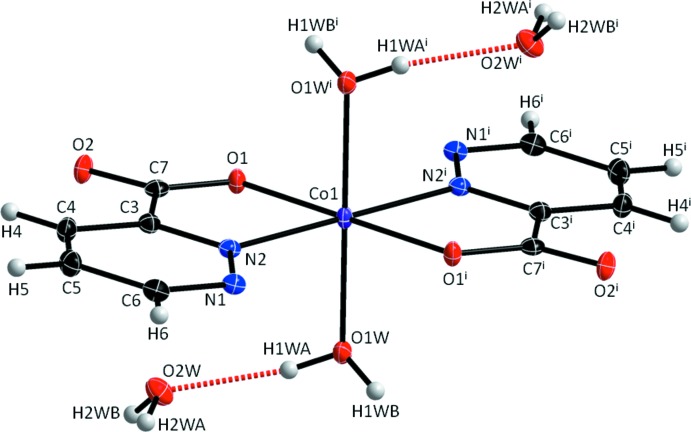
Molecular structure of the title compound, showing atom labelling and 50% probability displacement ellipsoids. [Symmetry code: (i) 1 - x, 1 - y, 1 - z.]

**Fig. 2.**
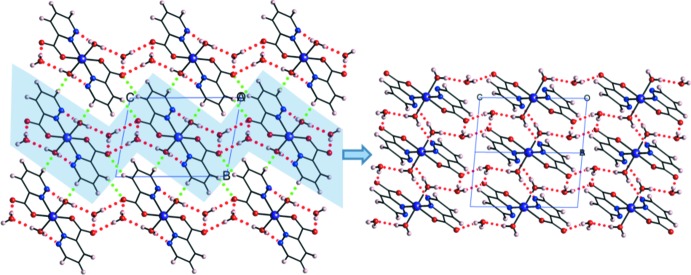
Left: View of the crystal packing along the crystallographic a axis. Right: Projection of a layer along the [110] direction (O—H···O and O—H···N hydrogen bonds represented as dotted red lines and C—H···O weak interactions as dotted green lines).

## supplementary crystallographic information

### Comment

The title compound, *trans*-[Co(C_4_H_3_N_2_O_2_)_2_(H_2_O)_2_].2H_2_O, crystallizes in the triclinic crystal system, space group P-1, and it is isostructural with the zinc and manganese complexes previously reported by Gryz *et al.* (2003) and Ardiwlnata *et al.* (1989). As expected, the Co—O and Co—N distances (Table 1) are similar to those of the Zn(II) and Mn(II) analogues. Table 2 summarizes the geometrical parameters of the O—H···O and N—H···O hydrogen bonding interactions.

### Experimental

To a solution of CoCl_2_.6H_2_O (71 mg, 0.3 mmol) in water (15 ml) 3-pyridazine carboxylic acid (74 mg, 0.6 mmol) was added and the resulting solution was stirred for 30 min at 90 °C. Prismatic orange crystals were obtained by slow evaporation after two days.

### Refinement

H atoms of the water molecules were located in a Fourier difference map and refined isotropically with O—H bond lengths restrained to 0.84 (2) Å. All H atoms of the pyridazine ring were positioned geometrically and refined using a riding model with C—H = 0.95 Å and *U*_iso_(H) = 1.2*U*_eq_(C).

### Crystal data

**Table:**

[Co(C_5_H_3_N_2_O_2_)_2_(H_2_O)_2_]·2H_2_O	*Z* = 1
*M**_r_* = 377.18	*F*(000) = 193
Triclinic, *P*1	*D*_x_ = 1.805 Mg m^−^^3^
Hall symbol: -P 1	Mo *K*α radiation, λ = 0.71073 Å
*a* = 5.2934 (4) Å	Cell parameters from 1404 reflections
*b* = 7.2817 (8) Å	θ = 2.2–27.8°
*c* = 9.6196 (9) Å	µ = 1.29 mm^−^^1^
α = 79.673 (8)°	*T* = 100 K
β = 89.875 (7)°	Prism, orange
γ = 72.321 (8)°	0.09 × 0.07 × 0.05 mm
*V* = 347.01 (6) Å^3^	

### Data collection

**Table:**

Agilent SuperNova Single source at offset diffractometer	1369 independent reflections
Radiation source: SuperNova (Mo) X-ray Source	1309 reflections with *I* > 2σ(*I*)
Mirror monochromator	*R*_int_ = 0.018
Detector resolution: 16.2439 pixels mm^-1^	θ_max_ = 26°, θ_min_ = 2.2°
ω scans	*h* = −6→6
Absorption correction: multi-scan (*CrysAlis PRO*; Agilent, 2011)	*k* = −8→7
*T*_min_ = 0.907, *T*_max_ = 0.967	*l* = −11→11
2202 measured reflections	

### Refinement

**Table:**

Refinement on *F*^2^	Primary atom site location: structure-invariant direct methods
Least-squares matrix: full	Secondary atom site location: difference Fourier map
*R*[*F*^2^ > 2σ(*F*^2^)] = 0.026	Hydrogen site location: difference Fourier map
*wR*(*F*^2^) = 0.058	H atoms treated by a mixture of independent and constrained refinement
*S* = 1.08	*w* = 1/[σ^2^(*F*_o_^2^) + (0.0165*P*)^2^ + 0.2394*P*] where *P* = (*F*_o_^2^ + 2*F*_c_^2^)/3
1369 reflections	(Δ/σ)_max_ < 0.001
122 parameters	Δρ_max_ = 0.28 e Å^−^^3^
4 restraints	Δρ_min_ = −0.31 e Å^−^^3^

### Special details

**Table:**

Experimental. IR (cm^-1^): 3500(*s*), 3320(*s*), 3229(*s*), 3075(*s*), 1626(*s*), 1580(*m*), 1559(*s*), 1451(w), 1385(w), 1227(w), 1163(w), 1090(w), 1074(w), 1034(w), 988(*m*), 851(*m*), 783(*m*), 721(*m*), 675(*m*), 536(w), 440(w).
Geometry. All e.s.d.'s (except the e.s.d. in the dihedral angle between two l.s. planes) are estimated using the full covariance matrix. The cell e.s.d.'s are taken into account individually in the estimation of e.s.d.'s in distances, angles and torsion angles; correlations between e.s.d.'s in cell parameters are only used when they are defined by crystal symmetry. An approximate (isotropic) treatment of cell e.s.d.'s is used for estimating e.s.d.'s involving l.s. planes.
Refinement. Refinement of *F*^2^ against ALL reflections. The weighted *R*-factor *wR* and goodness of fit *S* are based on *F*^2^, conventional *R*-factors *R* are based on *F*, with *F* set to zero for negative *F*^2^. The threshold expression of *F*^2^ > σ(*F*^2^) is used only for calculating *R*-factors(gt) *etc*. and is not relevant to the choice of reflections for refinement. *R*-factors based on *F*^2^ are statistically about twice as large as those based on *F*, and *R*- factors based on ALL data will be even larger.

### Fractional atomic coordinates and isotropic or equivalent isotropic displacement parameters (Å^2^)

**Table:**

	*x*	*y*	*z*	*U*_iso_*/*U*_eq_	
Co1	0.5	0.5	0.5	0.00870 (12)	
O1W	0.3333 (3)	0.3129 (2)	0.41228 (15)	0.0114 (3)	
N1	−0.0009 (3)	0.8482 (2)	0.40078 (17)	0.0106 (3)	
O1	0.7179 (3)	0.52521 (19)	0.32270 (14)	0.0108 (3)	
O2	0.7178 (3)	0.7107 (2)	0.11041 (14)	0.0134 (3)	
N2	0.2411 (3)	0.7466 (2)	0.36752 (17)	0.0097 (3)	
O2W	0.2458 (3)	0.4748 (2)	0.13080 (16)	0.0164 (3)	
C5	−0.0299 (4)	1.0868 (3)	0.1892 (2)	0.0143 (4)	
H5	−0.1268	1.2074	0.1306	0.017*	
C7	0.6162 (4)	0.6691 (3)	0.2244 (2)	0.0105 (4)	
C3	0.3458 (4)	0.8063 (3)	0.2481 (2)	0.0098 (4)	
C6	−0.1312 (4)	1.0140 (3)	0.3137 (2)	0.0123 (4)	
H6	−0.3022	1.0865	0.3375	0.015*	
C4	0.2144 (4)	0.9783 (3)	0.1543 (2)	0.0130 (4)	
H4	0.2908	1.0191	0.0695	0.016*	
H2WA	0.086 (3)	0.541 (3)	0.122 (3)	0.029 (7)*	
H1WA	0.291 (5)	0.359 (3)	0.3285 (18)	0.024 (7)*	
H2WB	0.281 (5)	0.420 (4)	0.062 (2)	0.032 (8)*	
H1WB	0.214 (4)	0.281 (4)	0.454 (3)	0.031 (7)*	

### Atomic displacement parameters (Å^2^)

**Table:**

	*U*^11^	*U*^22^	*U*^33^	*U*^12^	*U*^13^	*U*^23^
Co1	0.0088 (2)	0.0093 (2)	0.00666 (19)	−0.00195 (15)	0.00114 (14)	0.00030 (14)
O1W	0.0119 (7)	0.0136 (7)	0.0087 (7)	−0.0048 (6)	0.0012 (6)	−0.0001 (6)
N1	0.0095 (8)	0.0113 (8)	0.0114 (8)	−0.0029 (7)	0.0008 (6)	−0.0036 (7)
O1	0.0104 (7)	0.0111 (7)	0.0088 (7)	−0.0019 (6)	0.0010 (5)	0.0012 (5)
O2	0.0161 (7)	0.0143 (7)	0.0089 (7)	−0.0045 (6)	0.0051 (6)	−0.0002 (6)
N2	0.0091 (8)	0.0116 (8)	0.0096 (8)	−0.0040 (7)	0.0008 (6)	−0.0035 (7)
O2W	0.0148 (8)	0.0210 (8)	0.0127 (8)	−0.0029 (7)	0.0002 (6)	−0.0060 (6)
C5	0.0163 (10)	0.0095 (10)	0.0146 (11)	−0.0021 (8)	−0.0025 (8)	0.0009 (8)
C7	0.0114 (9)	0.0104 (9)	0.0119 (10)	−0.0053 (8)	0.0002 (8)	−0.0046 (8)
C3	0.0103 (9)	0.0105 (9)	0.0097 (9)	−0.0037 (8)	0.0009 (7)	−0.0037 (8)
C6	0.0106 (10)	0.0127 (10)	0.0138 (10)	−0.0024 (8)	0.0005 (8)	−0.0049 (8)
C4	0.0160 (10)	0.0129 (10)	0.0102 (10)	−0.0057 (8)	0.0012 (8)	0.0002 (8)

### Geometric parameters (Å, º)

**Table:**

Co1—O1	2.0689 (13)	O2—C7	1.249 (2)
Co1—O1^i^	2.0689 (13)	N2—C3	1.334 (2)
Co1—N2^i^	2.1023 (16)	O2W—H2WA	0.835 (17)
Co1—N2	2.1023 (16)	O2W—H2WB	0.824 (17)
Co1—O1W	2.1199 (14)	C5—C4	1.372 (3)
Co1—O1W^i^	2.1199 (14)	C5—C6	1.395 (3)
O1W—H1WA	0.819 (16)	C5—H5	0.95
O1W—H1WB	0.822 (17)	C7—C3	1.520 (3)
N1—C6	1.330 (2)	C3—C4	1.391 (3)
N1—N2	1.341 (2)	C6—H6	0.95
O1—C7	1.259 (2)	C4—H4	0.95
			
O1—Co1—O1^i^	180	C3—N2—N1	121.11 (16)
O1—Co1—N2^i^	101.76 (6)	C3—N2—Co1	113.96 (13)
O1^i^—Co1—N2^i^	78.24 (6)	N1—N2—Co1	124.73 (12)
O1—Co1—N2	78.24 (6)	H2WA—O2W—H2WB	108 (3)
O1^i^—Co1—N2	101.76 (6)	C4—C5—C6	117.74 (18)
N2^i^—Co1—N2	180	C4—C5—H5	121.1
O1—Co1—O1W	89.54 (5)	C6—C5—H5	121.1
O1^i^—Co1—O1W	90.46 (5)	O2—C7—O1	126.21 (18)
N2^i^—Co1—O1W	89.58 (6)	O2—C7—C3	117.09 (17)
N2—Co1—O1W	90.42 (6)	O1—C7—C3	116.69 (16)
O1—Co1—O1W^i^	90.46 (5)	N2—C3—C4	122.10 (18)
O1^i^—Co1—O1W^i^	89.54 (5)	N2—C3—C7	114.00 (16)
N2^i^—Co1—O1W^i^	90.42 (6)	C4—C3—C7	123.89 (17)
N2—Co1—O1W^i^	89.58 (6)	N1—C6—C5	123.38 (18)
O1W—Co1—O1W^i^	180.00 (4)	N1—C6—H6	118.3
Co1—O1W—H1WA	109.3 (17)	C5—C6—H6	118.3
Co1—O1W—H1WB	117.4 (18)	C5—C4—C3	117.37 (18)
H1WA—O1W—H1WB	112 (2)	C5—C4—H4	121.3
C6—N1—N2	118.24 (16)	C3—C4—H4	121.3
C7—O1—Co1	116.67 (12)		
			
N2^i^—Co1—O1—C7	−176.98 (13)	Co1—O1—C7—C3	−0.1 (2)
N2—Co1—O1—C7	3.02 (13)	N1—N2—C3—C4	1.8 (3)
O1W—Co1—O1—C7	93.54 (13)	Co1—N2—C3—C4	−173.40 (14)
O1W^i^—Co1—O1—C7	−86.46 (13)	N1—N2—C3—C7	−177.51 (15)
C6—N1—N2—C3	−2.1 (3)	Co1—N2—C3—C7	7.27 (19)
C6—N1—N2—Co1	172.59 (13)	O2—C7—C3—N2	175.85 (16)
O1—Co1—N2—C3	−5.73 (12)	O1—C7—C3—N2	−5.0 (2)
O1^i^—Co1—N2—C3	174.27 (12)	O2—C7—C3—C4	−3.5 (3)
O1W—Co1—N2—C3	−95.18 (13)	O1—C7—C3—C4	175.72 (17)
O1W^i^—Co1—N2—C3	84.82 (13)	N2—N1—C6—C5	0.4 (3)
O1—Co1—N2—N1	179.25 (15)	C4—C5—C6—N1	1.5 (3)
O1^i^—Co1—N2—N1	−0.75 (15)	C6—C5—C4—C3	−1.7 (3)
O1W—Co1—N2—N1	89.80 (14)	N2—C3—C4—C5	0.2 (3)
O1W^i^—Co1—N2—N1	−90.20 (14)	C7—C3—C4—C5	179.45 (17)
Co1—O1—C7—O2	179.04 (15)		

Symmetry code: (i) −*x*+1, −*y*+1, −*z*+1.

### Hydrogen-bond geometry (Å, º)

**Table:**

*D*—H···*A*	*D*—H	H···*A*	*D*···*A*	*D*—H···*A*
O2*W*—H2*WA*···O2^ii^	0.83 (2)	1.96 (2)	2.787 (2)	175 (2)
O1*W*—H1*WA*···O2*W*	0.82 (2)	1.92 (2)	2.732 (2)	171 (3)
O2*W*—H2*WB*···O2^iii^	0.83 (2)	2.05 (2)	2.865 (2)	168 (3)
O1*W*—H1*WB*···N1^iv^	0.82 (2)	2.07 (3)	2.862 (2)	164 (3)
C4—H4···O2^v^	0.95	2.37	3.188 (2)	145
C6—H6···O1*W*^vi^	0.95	2.33	3.264 (3)	166

Symmetry codes: (ii) *x*−1, *y*, *z*; (iii) −*x*+1, −*y*+1, −*z*; (iv) −*x*, −*y*+1, −*z*+1; (v) −*x*+1, −*y*+2, −*z*; (vi) *x*−1, *y*+1, *z*.
